# Deregulation of Hepatic Mek1/2–Erk1/2 Signaling Module in Iron Overload Conditions

**DOI:** 10.3390/ph12020070

**Published:** 2019-05-07

**Authors:** Naveen Kumar Tangudu, Nils Buth, Pavel Strnad, Ion C. Cirstea, Maja Vujić Spasić

**Affiliations:** 1 Institute of Comparative Molecular Endocrinology, University of Ulm, Ulm 89081, Germany; naveen.tangudu@uni-ulm.de (N.K.T.); nils.buth@uni-ulm.de (N.B.); ion.cirstea@uni-ulm.de (I.C.C.); 2 Department of Medicine III and IZKF, University Hospital Aachen, Aachen 52074, Germany; pstrnad@ukaachen.de

**Keywords:** liver, iron, hepcidin, Mek/Erk, Hfe, Bmp/Smad

## Abstract

The liver, through the production of iron hormone hepcidin, controls body iron levels. High liver iron levels and deregulated hepcidin expression are commonly observed in many liver diseases including highly prevalent genetic iron overload disorders. In spite of a number of breakthrough investigations into the signals that control hepcidin expression, little progress has been made towards investigations into intracellular signaling in the liver under excess of iron. This study examined hepatic signaling pathways underlying acquired and genetic iron overload conditions. Our data demonstrate that hepatic iron overload associates with a decline in the activation of mitogen-activated protein kinase (MAPK)/extracellular signal-regulated kinase (Erk) kinase (Mek1/2) pathway by selectively affecting the phosphorylation of Erk1/2. We propose that Mek1/2-Erk1/2 signaling is uncoupled from iron-Bmp-Smad-mediated hepcidin induction and that it may contribute to a number of liver pathologies in addition to toxic effects of iron. We believe that our findings will advance the understanding of cellular signaling events in the liver during iron overload of different etiologies.

## 1. Introduction

In vertebrates, the liver is an essential metabolic hub. It hosts numerous biochemical processes and regulates the storage of many essential nutrients, vitamins, and minerals, as well as their release when there is a physiological need for them. The liver is the main parenchymal iron repository and at the same time the principal organ controlling systemic iron fluxes through the production of the iron hormone hepcidin [1]. High hepatic iron levels are commonly observed in many liver diseases including highly prevalent genetic iron overload disorders (i.e., hereditary hemochromatosis), hematologic disorders (i.e., thalassemia and sickle cell disease), chronic hepatitis C, alcoholic liver disease and non-alcoholic fatty liver disease l [2,3]. The main pathological effects of hepatic iron overload include liver fibrosis, cirrhosis and hepatocellular carcinoma.

The sensing of plasma iron levels (i.e., transferrin-bound iron) by liver hepatocytes involves a multiprotein membrane-bound complex consisting of transferrin receptors 1 and 2 (TfR1, TfR2), MHC I-like protein Hfe, hemojuvelin, and the bone morphogenetic protein (Bmp) receptors type I such as activin receptor-like kinase 1 (Alk2/Acvr1) and Alk3/Bmpr1a [4,5,6,7,8,9,10,11,12]. The Bmp-Alk2/3-Smad1/5/8 cascade is currently considered as the central intracellular relay communicating high plasma iron levels to hepcidin, since mice and patients with genetic disruption in the iron-sensing molecules show impaired Bmp/Smad signaling, low hepcidin expression and consequently develop hepatic iron overload [12,13,14,15]. Furthermore, the contribution of the extracellular signal-regulated kinases 1 and 2 (Erk1/2) to hepcidin regulation was proposed: studies in erythroleukemia K562 cells, exposed to holo-transferrin, demonstrated activation of ERK1/2 and p38 MAP kinases, a process that was dependent on TfR2 [16]; consequently, silencing of TfR2 and Hfe in HepG2 cells and in mice resulted in decreased ERK1/2 signaling and low hepcidin expression in the liver [8,17]. It was proposed that ERK1/2 might act in concert with Bmp-Smad signaling to control hepcidin expression [18].

The mitogen-activated protein kinases (MAPKs) are among the largest protein families in eukaryotes that transduce a variety of extracellular signals to regulate a plethora of cellular responses [19,20]. MAPKs consist of many protein kinases but three major protein kinases are extensively studied: the ERK1/2, activated by broad spectrum of extracellular ligands such as mitogens/growth factors and differentiation signals [21], and c-Jun amino terminal kinases (JNK1/2/3) and p38 kinases that are activated by stress stimuli [19,20,22]. MAPK-dependent signal transduction is required to maintain physiological metabolic adaptation while inappropriate MAPK signaling has been increasingly associated with the development of metabolic syndrome [23]. In the liver, the MAPKs play an important role in processes that regulate metabolism [23,24,25,26]. In particular, activation of stress-responsive p38MAPKs and JNKs was associated with hepatic metabolic dysfunction [20,23,26,27,28], whereas constitutive Erk1 or liver-specific Erk2 deficiency in mice was proposed to affect hepatic glucose and lipid metabolism, promote insulin resistance and ER stress [27,29,30].

In this study, we examined the association between hepatic iron overload in mice, caused by parenteral, nutritional and genetic iron overload, and the activity of Mek1/2-Erk1/2 signaling. Our data demonstrate decreased Mek1/2-Erk1/2 signaling output in iron overloaded conditions, suggesting that Mek1/2-Erk1/2 signaling is uncoupled from Bmp-Smad1/5/8-mediated hepcidin induction and that it may play an important role in liver diseases characterized by hepatic iron excess. 

## 2. Results and Discussion

### 2.1. Classical and Stress-Induced MAPKs Activation in Iron-Loaded Livers

**Figure 1:**
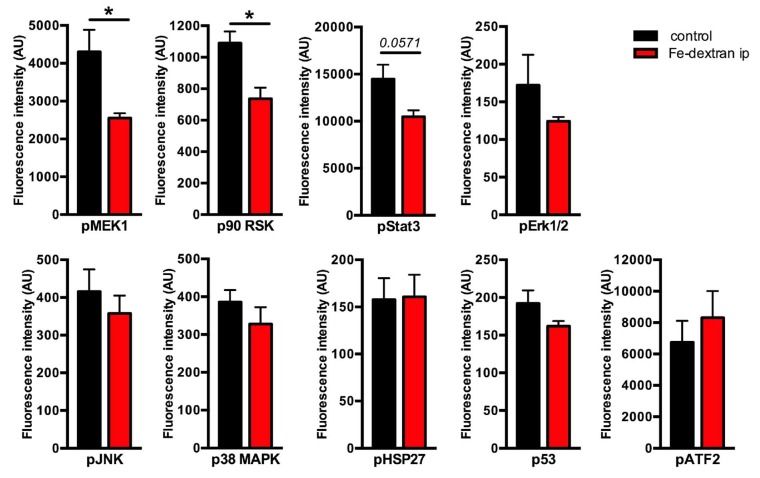
Identification of activated proteins in iron overloaded livers by Bio-Plex Pro Cell Signaling MAPK Panel 9-plex. Phosphorylation status of nine intracellular phosphoproteins was measured in the livers of iron-dextran injected mice and compared to controls. Data were analyzed using GraphPad Prism software and results are shown as mean ± SEM (standard error of mean). For the statistical analysis, a nonparametric distribution and the unpaired Mann–Whitney U Test were used. * *p*-values < 0.05; AU: arbitrary units; n = 4; 4 per group.

The aim of this study was to investigate the activity of intracellular signaling pathways in the livers under excessive iron overload. To this end, we used our previously established mouse model of parenteral iron overload, which is characterized by severe hepatic iron overload, high circulating iron levels, and increased hepcidin mRNA expression [15]. We measured the phosphorylation status of nine intracellular proteins including Mek1 (Ser217/Ser221) and Erk1/2 (Thr202/Tyr204, Thr185/Tyr187), as classical MAPK backbone components, stress-responsive MAPKs, such as JNK (Thr183/Tyr185) and p38 MAPK (Thr180/Tyr182), and effector molecules of MAPKs including p90 RSK (Ser380), Stat3 (Ser727), ATF-2 (Thr71), HSP27 (Ser78), and p53 (Ser15), using Bio-Plex Pro Cell Signaling MAPK Panel 9-plex (BioRad Laboratories, Germany). This analysis revealed a decrease in the phosphorylation levels of Mek1 and p90 RSK in iron-loaded livers, and only marginally in case of Stat3 (*p* < 0.0571); similarly, the levels of pErk1/2 showed a trend towards a decrease however the data were under the level of statistical significance (Figure 1). The levels of pJNK, p38MAPK, pHSP27, p53, and pATF-2 showed no statistically significant differences (Figure 1). 

Based on these results, we postulated that the presence of hepatic iron overload might associate with selective impairment of the Mek1/2-Erk1/2 pathway and its downstream pStat3 and pp90 RSK targets. This idea is supported by recent investigations showing selective activation of JNK and the p38 MAPK signaling activity under cellular iron depletion [31]. Moreover, in response to growth factors, Erk1/2, but not JNK or p38, specifically phosphorylate Stat3 at Ser727, which is also stimulated by interleukine-6 cytokine, however, in contrast to growth factors, the latter process occurs independent of Erk1/2 [32]. 

### 2.2. Association of Mitogen-activated Protein Kinases (MAPK) Activity with Hepatic Iron Overload

**Figure 2:**
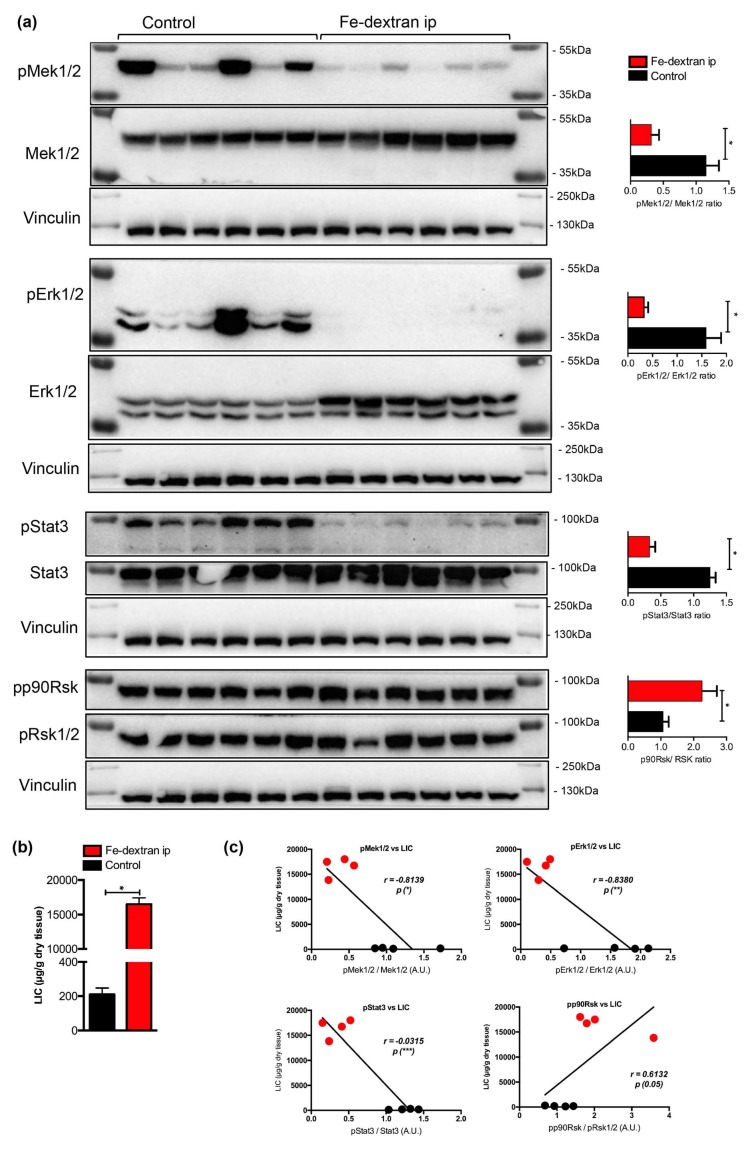
(**a**) Immunoblot analysis and relative quantification (shown in histograms on right) of pMEK1, pERK1/2, pStat3, and pp90Rsk in the livers of iron-dextran injected and control mice (n = 6; 6 mice per group). (**b**) Liver iron content (LIC) of control and iron-dextran injected mice. (**c**) Correlation analysis between LIC and the levels of pMek1/2/Mek1/2, pErk1/2/Erk1/2, pStat3/Stat3, and pp90Rsk/pRsk1/2 in the livers of iron-dextran injected and control mice. M: Page Ruler Plus Prestained Protein Ladder (Thermo Scientific). Data were analyzed using GraphPad Prism software and results are shown as mean ± SEM (standard error of mean). For the statistical analysis, a nonparametric distribution and the unpaired Mann–Whitney U Test were used. Linear regression and Pearson correlation coefficients were computed for every data set with 95% confidence intervals. * *p* values < 0.05.

To test the above hypothesis we evaluated phosphorylation levels of Mek1/2, Erk1/2, Stat3 and p90 Rsk proteins in iron-loaded livers using immunoblotting analysis. This analysis revealed a significant decrease in the phosphorylation levels of Mek1/2, Erk1/2, and Stat3 (by 3.6-, 4.8-, and 3.8-fold, respectively), while the levels of p90Rsk were increased by 2.1-fold (Figure 2a). 

### 2.3. Nutritional Iron Overload is Characterized by Low Hepatic Mek1/2-Erk1/2-Stat3 Signaling

**Figure 3:**
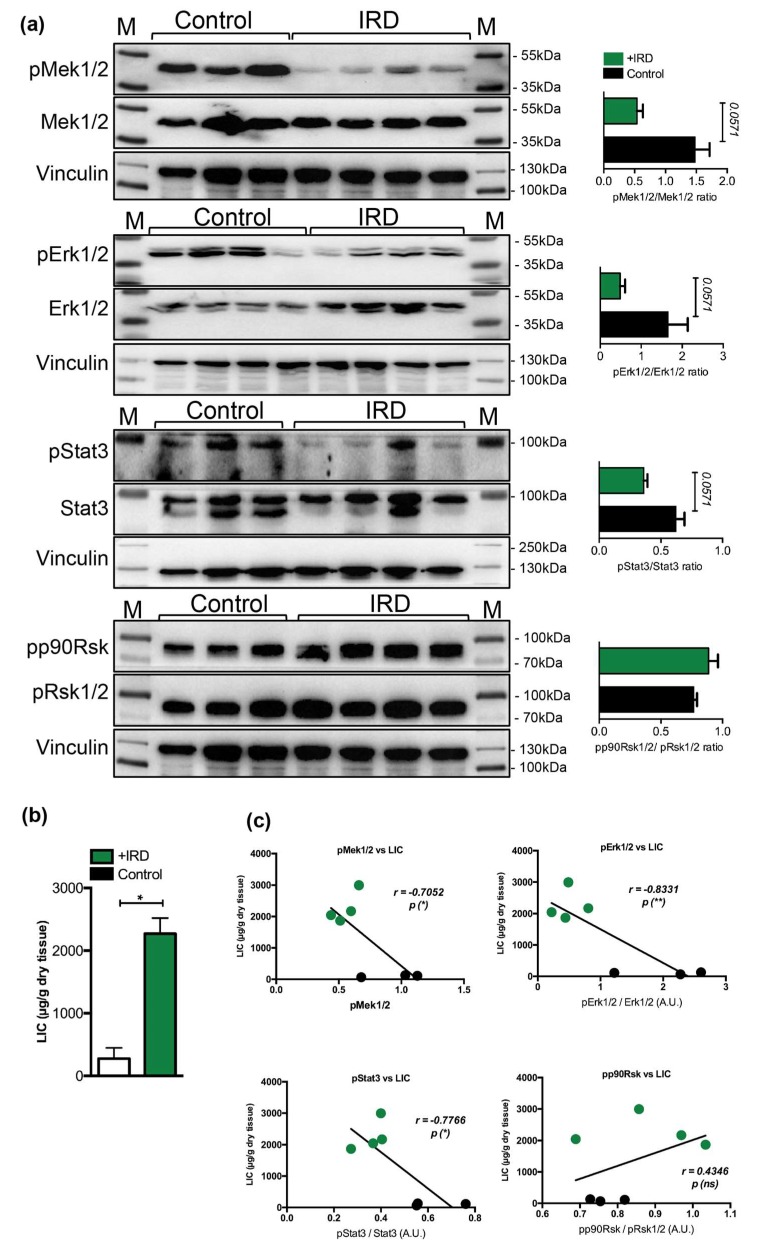
(**a**) Representative immunoblot analysis and relative quantification (shown in histograms on right) of pMek1/2, pErk1/2, pStat3, and pp90Rsk in the livers of mice maintained on an iron rich diet (IRD) and standard diet (control) (n = 3–4 mice per group). (**b**) Liver iron content (LIC) of control and of mice maintained on an iron rich diet (n = 3–4 mice per group). (**c**) Correlation analysis between LIC and the levels of pMek1/2/Mek1/2, pErk1/2/Erk1/2, pStat3/Stat3, and pp90Rsk/pRsk1/2 in the livers of mice maintained on an iron rich diet (IRD) and standard diet (control) (n = 3–4 mice per group). M: Page Ruler Plus Prestained Protein Ladder (Thermo Scientific). Data were analyzed using GraphPad Prism software and results are shown as mean ± SEM (standard error of mean). For the statistical analysis, a nonparametric distribution and the unpaired Mann–Whitney U Test were used. Linear regression and Pearson correlation coefficients were computed for every data set with 95% confidence intervals. * *p*-values < 0.05.

We next questioned whether a decrease in the Mek1/2-Erk1/2-Stat3 activity, measured during parenteral iron overload, is present in conditions of nutritional iron overload. We thus examined the phosphorylation status of Mek1/2-Erk1/2-Stat3 in the livers of wild type mice undergoing nutritional iron overload, induced by feeding mice with 3% carbonyl iron containing diet for six months starting at one month of age, as we previously described [33]. We show that hepatic iron overload, induced by iron-rich diet, associated with a 2.7-, 3.4-, and 1.7-fold decrease in the phosphorylation levels of Mek1/2-Erk1/2-Stat3 proteins, respectively, while the levels of p90Rsk were unchanged (Figure 3). However the data were only marginally under the level of statistical significance (*p* < 0.0571), which is, in our opinion, caused by stringent statistical analysis we applied (unpaired, nonparametric, Mann–Whitney test; n = 3–4 mice per group). Collectively, our data imply that hepatic response of mice receiving parenteral or dietary iron overload associates with a selective decline in the activity of Mek1/2-Erk1/2-Stat3 branch.

Future studies are needed to determine whether a decrease in phosphorylation of Mek1/2-Erk1/2 might be caused by diminished tyrosine kinase receptors (such as epidermal growth factor receptor, EGFR) mediated signaling activity and whether its activity may correlate with changes in the levels of extracellular ligands that can bind EGFR, such as EGF, transforming growth factor alpha, amphiregulin, heparin-binding EGF, and others. In addition, it will be informative to measure the levels of growth hormone (GH), as defective GH signaling impairs activation of EGFR and ERK signaling and affects liver regeneration [34]. 

### 2.4. Low Hepatic Mek1/2-Erk1/2 Signaling is Present in Hfe-/- Mice in Spite of Low Bmp-Smad Signaling

**Figure 4:**
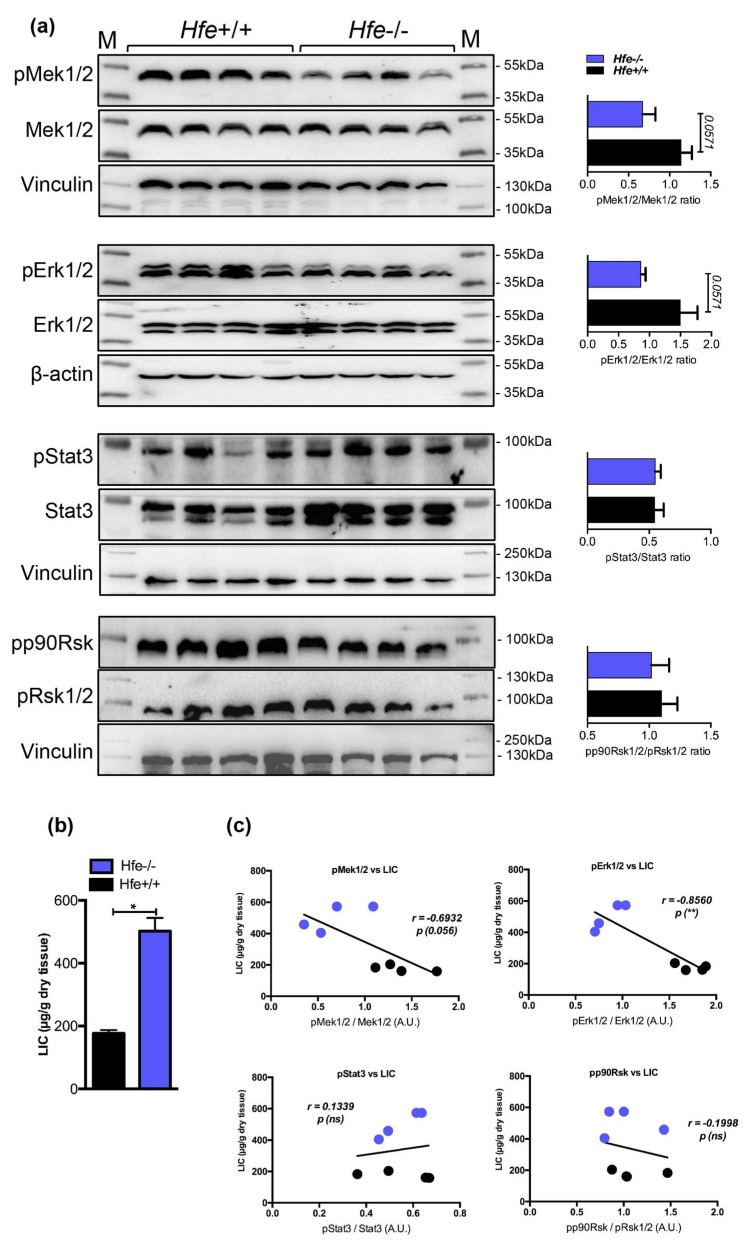
(**a**) Representative immunoblot analysis and relative quantification (shown in histograms on right) of pMek1/2, pErk1/2, pStat3 and pp90Rsk in the livers of Hfe+/+ control and Hfe-/- mutant mice (n = 8;10 mice per group). (**b**) Liver iron content (LIC) of Hfe+/+ and Hfe-/- mice (n = 4;4 mice per group). (**c**) Correlation analysis between LIC and the levels of pMek1/2/Mek1/2, pErk1/2/Erk1/2, pStat3/Stat3, and pp90Rsk/pRsk1/2 in the livers of Hfe+/+ and Hfe-/- mice (n = 4;4 mice per group). M: Page Ruler Plus Prestained Protein Ladder (Thermo Scientific). Data were analyzed using GraphPad Prism software and results are shown as mean ± SEM (standard error of mean). For the statistical analysis, a nonparametric distribution and the unpaired Mann–Whitney U Test were used. Linear regression and Pearson correlation coefficients were computed for every data set with 95% confidence intervals. * *p*-values <0.05.

So far, our data indicate a decrease in pMek1/2-pErk1/2-pStat3 signaling under excessive systemic and hepatic iron overload. Contrary to Mek1/2-Erk1/2, the activity of Bmp-Smad signaling was shown to increase following iron overload subsequently causing increase in the expression of hepcidin and genes known to be coregulated with hepcidin [15]. Given that previous studies suggest co-involvement of Erk1/2 and Bmp/Smad signaling for induction of hepcidin in cells [18], it seemed logical to investigate the Mek1/2-Erk1/2 signaling activity in conditions characterized by hepatic iron overload and low hepcidin expression. To this end, we employed *Hfe*-deficient mice, a well-established mouse model of genetic iron-overload, which due to the lack of Hfe, an upstream hepcidin regulator, showed impaired Bmp-Smad signaling, low hepcidin expression, and increased hepatic iron stores [13,14,15]. We detected that the activation of Mek1/2-Erk1/2, measured by phosphorylation status using immunoblot analysis, was on average 2-fold lower in the livers of *Hfe*-/- mice (with marginal significance of *p* = 0.0571), whereas the levels of pStat3 and pp90Rsk were not significantly changed (Figure 4). These data suggest that a decrease in Mek1/2-Erk1/2 signaling is uncoupled from Bmp-Smad signaling activity and Smad-mediated hepcidin regulation.

### 2.5. Low Hepatic Mek1/2-Erk1/2 Signaling is Present in Hepcidin-Deficient Mice and is Further Aggravated by Iron Excess

**Figure 5:**
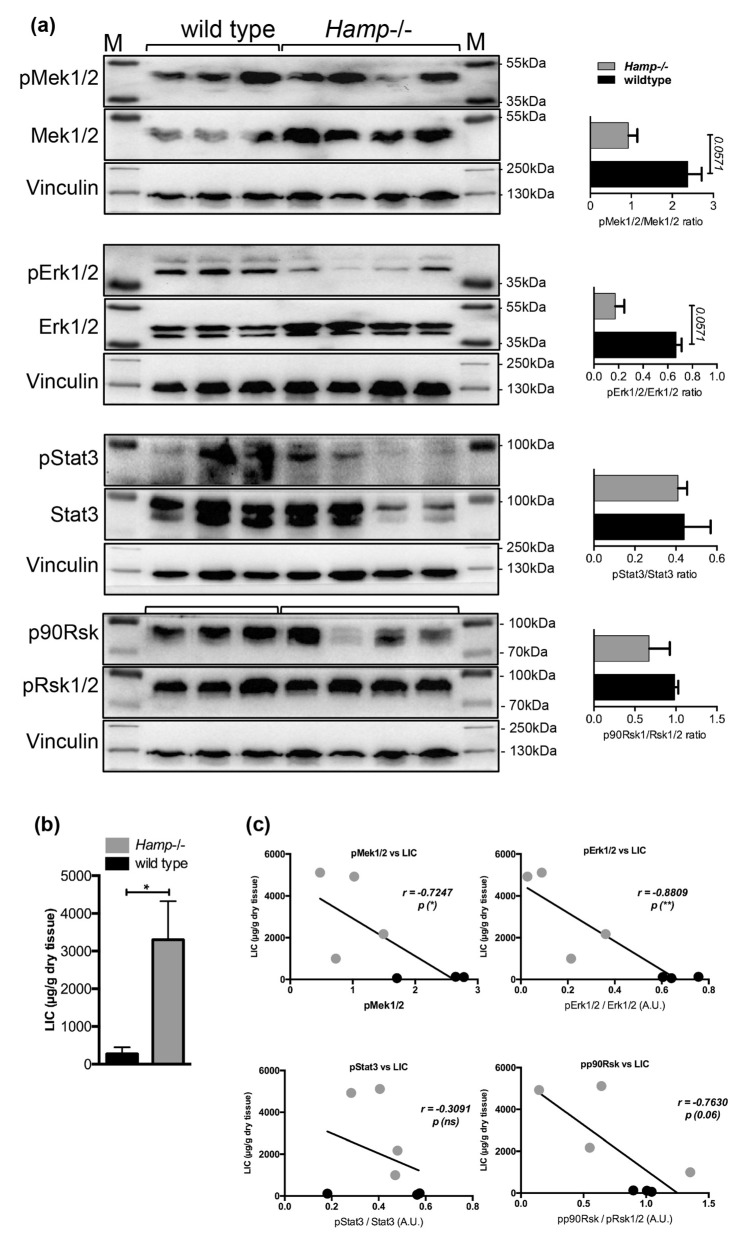

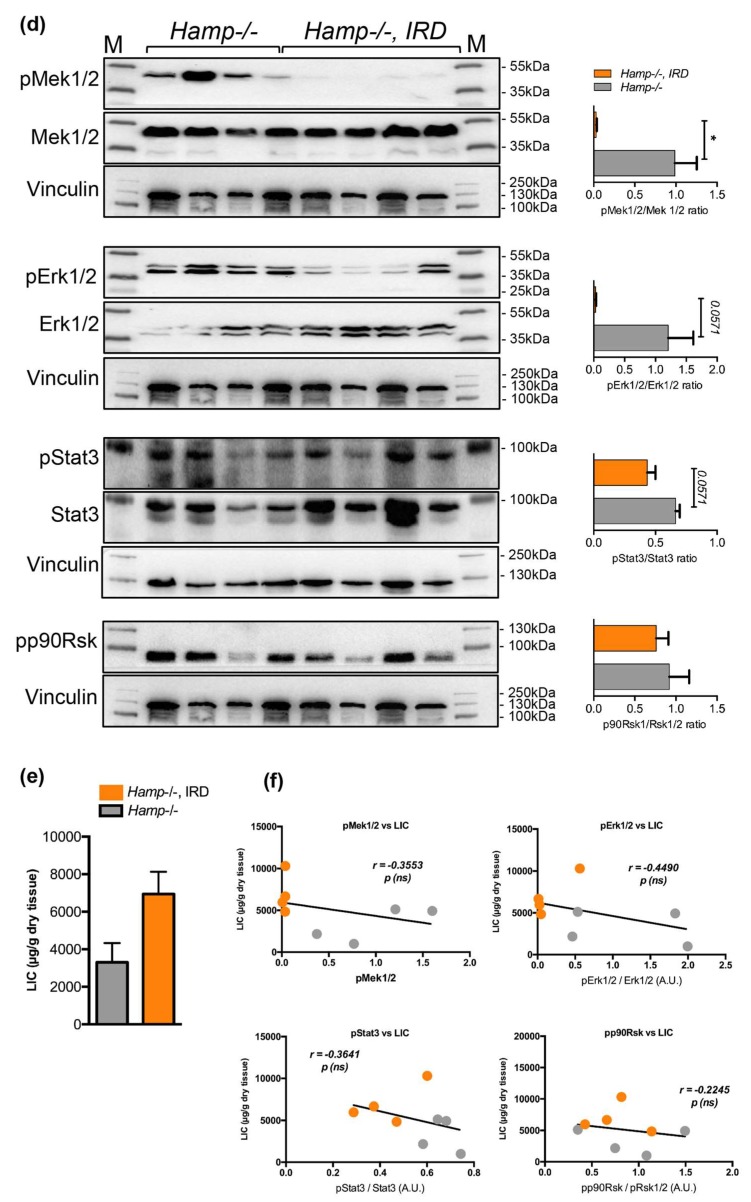
Immunoblot analysis and relative quantification (shown in histograms on right) of pMek1/2, pErk1/2, pStat3 and pp90Rsk in the livers of (**a**) control and Hamp-/- mice and (**d**) Hamp-/- mice maintained on an iron-rich diet (IRD) (n = 3;4;4 mice per group). Liver iron content (LIC) in (**b**) control, Hamp-/- and (**e**) Hamp-/- mice maintained on an iron-rich diet (n = 3;4;4 mice per group). Correlation analysis between LIC and the levels of pMek1/2/Mek1/2, pErk1/2/Erk1/2, pStat3/Stat3, and pp90Rsk/pRsk1/2 in the livers of (**c**) control, Hamp-/- and (**f**) Hamp-/- mice maintained on an iron rich diet (IRD) (n = 3; 4;4 mice per group). M: Page Ruler Plus Prestained Protein Ladder (Thermo Scientific). Data were analyzed using GraphPad Prism software and results are shown as mean ± SEM (standard error of mean). For the statistical analysis, a nonparametric distribution and the unpaired Mann–Whitney U Test were used. Linear regression and Pearson correlation coefficients were computed for every data set with 95% confidence intervals. * *p*-values <0.05.

We next investigated Mek1/2-Erk1/2 activation status in the livers of hepcidin knock-out mice (*Hamp*-/-), which are deficient for hepcidin expression but maintain an appropriate Bmp-Smad signaling activity as a response to liver iron overload [15]. Similarly to the observation in the livers of *Hfe*-/- mice, a 2.5- and 3.8-fold decrease in the levels of pMek1/2-pErk1/2 proteins were measured in the livers of *Hamp*-/- mice (with marginal significance of *p* = 0.0571), while the pStat3 and pp90Rsk levels were not significantly changed (Figure 5a–c). Interestingly, the activation of Mek1/2, Erk1/2, and Stat3, measured by their phosphorylated levels, was suppressed by 34-, 7.5-, and 1.5-fold, respectively (with marginal significance of *p* = 0.0571), while the levels of pp90Rsk were unchanged in *Hamp*-/- mice fed an iron-rich diet for six months (Figure 5d–f), which caused the development of chronic liver injury as we previously demonstrated [33]. 

Interestingly, the initially observed increase in pp90Rsk levels following iron-dextran injections in mice (Figure 2a,c) was not detected in the livers of mice maintained on an iron-rich diet nor in Hfe-/- or Hamp-/- mice. A possible explanation might be the differences in the route of iron application (i.p injections versus nutritional/enteral iron administration) and the duration of iron loading (3-weeks of iron-dextran injections versus six months of iron-rich diet). We suspect that fast influx of iron and excessive liver iron loading following i.p. iron-dextran injections might differentially affect cellular and humoral responses than nutritional iron administration or genetic iron overload. 

Taken together, our data reinforce the view that attenuation of Mek1/2-Erk1/2 phosphorylation is a function of hepatic iron overload. Given the variety of iron-overload models used in this study, we speculate that changes in Mek1/2-Erk1/2 signaling may be categorized as initiating mechanism predisposing liver cells to toxic insults. Among them, inflammation, hepatic oxidative stress including the production of highly reactive lipid peroxidation products and iron-catalyzed oxidant stress [35,36] could ultimately lead towards the progression of a chronic liver disease. This hypothesis is supported by recent studies demonstrated that inhibition of ERK1/2 signaling sensitized the cells to chemotaxic stimuli [37]. 

**Figure 6:**
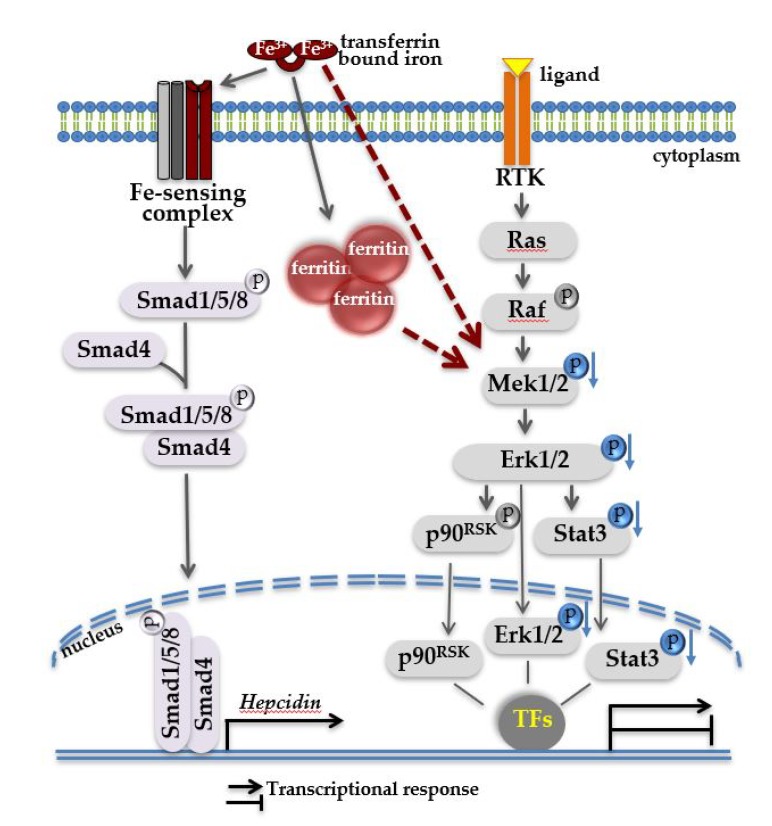
Proposed model of two signaling pathways operating in iron-loaded livers. Systemic iron overload results in heavy iron deposition in the liver, illustrated here in form of high ferritin. Under these conditions, high levels of circulating transferrin-bound iron is sensed by cell membrane multiprotein iron-sensing complex, resulting in the activation of intracellular Bmp-Smad signaling cascade and increased hepcidin transcription in the nucleus. Independent of Bmp-Smad-mediated hepcidin activation, through a yet unknown mechanism (proposed here by red arrows), a decrease in phosphorylation of Mek1/2-Erk1/2-Stat3 (indicated in blue) occurs which in turn may affect the property of pErk1/2 and pStat3 signaling molecules to regulate gene transcription, alone or in cooperation with other transcription factors (TFs). A decrease in the activity of Mek1/2-Erk1/2 signaling cascade can be considered as liver response to iron overload. We propose that a decrease in Mek1/2-Erk1/2 signaling activity may accelerate liver pathologies in addition to toxic effects of iron.

The collective data let to a working model (Figure 6), in which high hepatic iron burden associates with a decrease in phosphorylation levels of Mek1/2-Erk1/2. This in turn may contribute to a number of liver pathologies in addition to toxic effects of iron. 

Data presented in this study raise a number of questions as to whether (i) low Mek1/2-Erk1/2 activity occurs as a selective response to high hepatic iron levels or is triggered by high transferrin and/or non-transferrin-bound iron, (ii) in which hepatic cells (hepatocytes or nonparenchymal cells) and in which cellular compartments does Erk1/2 function, (iii) what is the level of Mek1/2-Erk1/2 cross‑talk with parallel pathways such as the PI3K/AKT/mTOR pathway in iron-loaded livers, and (iv) what are the consequences of inhibition of the Erk1/2 pathway in iron-loaded livers on hepatic gene transcription? 

Finally, our findings may be of relevance to other conditions where hepatic iron levels are increased such as alcoholic liver disease, characterized by low hepcidin expression, suppressed hepatic Erk1/2 activity and liver injury [27,38,39,40,41,42,43]. In addition, hepatic iron overload is present in approximately one-third of patients with nonalcoholic fatty liver disease [44,45,46,47], which is recognized as the most common chronic liver disease that can progress to non-alcoholic steatohepatitis and liver cancer [48,49,50]. Hepatic iron overload is also observed in patients with chronic hepatitis C virus infections (and rarely in chronic hepatitis B infections) and in end-stage liver disease [3,46,47,51,52,53,54]. Whether hepatic iron overload and the presence of suppressed Mek1/2-Erk1/2 signaling may either accelerate disease progression or whether maintaining low Mek1/2-Erk1/2 signaling may be protective from the induction of *c-Myc* and *c-Jun* as a part of increased proliferation, and therefore reduce the incidence of liver damage, are certainly important questions to address in the future. An equally interesting aspect would be to monitor Mek1/2-Erk1/2 activation from early steps of iron overload until development of liver pathologies and establish whether the activity of MAPK module is accordingly modulated. Understanding the underlying mechanisms associated with hepatic iron overload and progressive liver failure may provide new modalities for therapeutic interventions. We believe that the data provided here will advance our understanding of cellular signaling events in the liver during iron overload of different etiologies.

## 3. Materials and Methods

### 3.1. Mice and Treatments

Wild type *Hfe-/-* and *Hamp*-/- mutant mice, all males, were kept under a standard mouse diet containing 180 mg/kg iron (Ssniff, Soest, Germany). For the analysis livers were used from previously described wild-type mice undergoing intra peritoneal (i.p.) injection of iron-dextran solution [15,33] and from *Hamp*-/- and wild type mice fed with 3% carbonyl iron (Sigma, Germany) for 6 months [15,33]. Animal experiments were approved and performed in accordance to the Ulm University Animal Care Committee and German Low for Welfare of laboratory animals in Baden-Württemberg, Germany (Project ID: 35/9185.81-3 / 972 / 1143).

### 3.2. Phosphoprotein Analysis in Liver Lysates

Protein lysates (10 µg in 50 µL) were prepared from liver tissues using Bio-Plex Cell Lysis Kit (BioRad Laboratories, Munich, Germany). The levels of intracellular phosphoproteins were measured using Bio-Plex Pro Cell Signaling MAPK Panel, 9-plex (BioRad Laboratories, Munich, Germany) according to the manufacturer’s instructions. The data were analyzed using Bio-Plex Manager 6.1 Software Package.

### 3.3. Protein Isolation and Immunoblot Analysis

Protein extracts were prepared from flash-frozen tissue after homogenization in RIPA lysis buffer (Incomplete RIPA buffer, 7× protease inhibitor cocktail, 200mM sodium orthovandate, 1M sodium fluoride, 100mM PMSF) as previously described [15]. Total proteins (30–50 µg) were subjected to Western blot analysis with the following antibodies; anti-pMek1/2, anti-Mek1/2, anti-pErk1/2, anti-Erk1/2, anti-pp90Rsk, anti-pRsk1/2, anti-pStat3, and anti-Stat3 (all rabbit, Cell Signaling Technology, MA, USA; 1:1000 concentration). Mouse anti-vinculin (Santa Cruz, CA, USA; 1:2000) and mouse anti-β-actin (Sigma Aldrich, Missouri, USA; 1:10,000) were used as loading controls. Furthermore, membranes were washed and incubated with anti-rabbit or anti-mouse (Invitrogen, CA, USA; 1:5000) horseradish peroxidase-conjugated antibody. Western blot images were acquired using EMD Millipore Luminata HRP chemiluminescence substrate (Millipore, MA, USA) and signal acquired in Bio-Rad chemiluminescence detector (Bio-Rad Laboratories, CA, USA). The signals were semiquantified using image J (ImageJ; www://rsb.info.nih.gov/ij/).

### 3.4. Statistical Analysis

Data were analyzed using GraphPad Prism software and results are shown as mean ± standard error of mean. For the statistical analysis, a nonparametric distribution and the Mann–Whitney U test were used. Linear regression and Pearson correlation coefficients were computed for every data set with 95% confidence intervals. Statistically significant differences are indicated as *p* ˂ 05 (*), *p* ˂ 01 (**), and *p* ˂ 005 (***).
